# Understanding Phishing Email Processing and Perceived Trustworthiness Through Eye Tracking

**DOI:** 10.3389/fpsyg.2020.01756

**Published:** 2020-07-28

**Authors:** John McAlaney, Peter J. Hills

**Affiliations:** Faculty of Science & Technology, Department of Psychology, Bournemouth University, Poole, United Kingdom

**Keywords:** phishing, eye tracking, social engineering, cybersecurity, email

## Abstract

Social engineering attacks in the form of phishing emails represent one of the biggest risks to cybersecurity. There is a lack of research on how the common elements of phishing emails, such as the presence of misspellings and the use of urgency and threatening language, influences how the email is processed and judged by individuals. Eye tracking technology may provide insight into this. In this exploratory study a sample of 22 participants viewed a series of emails with or without indicators associated with phishing emails, whilst their eye movements were recorded using a SMI RED 500 eye-tracker. Participants were also asked to give a numerical rating of how trustworthy they deemed each email to be. Overall, it was found that participants looked more frequently at the indicators associated with phishing than would be expected by chance but spent less overall time viewing these elements than would be expected by chance. The emails that included indicators associated with phishing were rated as less trustworthy on average, with the presence of misspellings or threatening language being associated with the lowest trustworthiness ratings. In addition, it was noted that phishing indicators relating to threatening language or urgency were viewed before misspellings. However, there was no significant interaction between the trustworthiness ratings of the emails and the amount of scanning time for phishing indicators within the emails. These results suggest that there is a complex relationship between the presence of indicators associated with phishing within an email and how trustworthy that email is judged to be. This study also demonstrates that eye tracking technology is a feasible method with which to identify and record how phishing emails are processed visually by individuals, which may contribute toward the design of future mitigation approaches.

## Introduction

Internet browsers, email systems and other socio-technical systems require input from individual users. Such systems may be designed in a way that aims to protect users and organizations from external attackers as much as is possible (Das et al., 2020). How successful they are in doing so is highly reliant on the user (Pfeffel et al., 2019). The user may not fully attend to the cues and prompts that the system provides them with to encourage them to use the system in a safe way (Miyamoto et al., 2015). Similarly, users may fail to detect and respond to potential threats, even if the system provides prominent indicators of these threats (Miyamoto et al., 2015). An example of this is phishing websites, links to which are sent to potential victims through phishing emails. Phishing emails are one form of social engineering, which refers to the use of manipulation and trickery to cause an individual to gain sensitive information or access to a system (Hadnagy, 2018). This type of attack has been described as the single biggest threat to cybersecurity (Salahdine and Kaabouch, 2019). Individuals who engage in cyber-crime do not need to possess programming or technological skills in order to be able to create phishing emails; software packages that can be used to create phishing emails can be downloaded online (McCalley et al., 2011).

This reliance on user engagement in many sociotechnical systems is potentially problematic. As is well established in psychological research people often do not fully processes all the information that is available to them in any given situation (Gigerenzer and Brighton, 2009). That is, people are not always rationale decision makers. Instead they make use of decision-making heuristics, a form of a mental shortcut, to come to a quick decision based on a limited number of cues. This is known as the cognitive miser approach and contrasts with the naïve scientist approach in which individuals make decisions based on a more comprehensive and thorough evaluation of the information available (Fiske and Taylor, 2013). It has been argued that people are motivated tacticians, in which whether they apply a cognitive miser or naïve scientist approach is in part determined by the urgency, perceived importance and complexity of the situation (Kruglanski, 1996). This strategy reflects limitations in how much information individuals can process at any one time. If we were to attempt to fully process all the information that is available in every situation, we encounter each day, as in the naïve scientist approach, then this would become extremely time consuming (Sweller, 1988). On the other hand, using the cognitive miser approach may be quicker and less cognitively demanding, but is at greater risk of error, as the individual is basing their decision on a limited number of factors. As such individuals switch between strategies based on which they think will be the most optimal for the situation they are facing, an approach which will not always necessarily be correct (Gigerenzer and Brighton, 2009). The tactics used by social engineers are often based on exploiting heuristics, by including elements that encourage the target to engage the cognitive miser approach and make quick, less analytical decisions (Hadnagy, 2018). Examples of this within the social engineering technique of phishing emails can include the use of language that contains emotive elements such as threat, urgency, or financial information (Hadnagy, 2018). However, research connecting information processing to the characteristics of phishing emails is lacking. To fully understand how an individual engages with aspects of a socio-technical system such as phishing emails it is necessary therefore to explore how much and what information they are processing.

One way in which this can be achieved is through eye trackers. Eye tracking technologies are used to measure an individual’s eye movements and in turn to determine what they are looking at. This is known as the point of regard and is an indication of where the individual’s attention lies. By measuring several factors such as duration of fixations (when the eyes are relatively stationary), the length of saccades (when the eyes move between areas of interest), number of regressions (where the eyes return to a previously fixated point) inferences can be made about much cognitive processing the individual is giving to any part of the stimulus. This information can be combined to explore the scanpath. This refers to the sequence of fixations and eye movements over an image. For example, an eye tracker may be used to determine the scanpath of an individual viewing a web page, which could provide information about the order in which the individual views different parts of the website. This approach has been used extensively in Human-Computer Interaction studies, such as to assess website usability (Cowen et al., 2002). Related technologies can also be used to measure pupil dilation and blink rate, which can measure cognitive overload and fatigue, respectively (Stern et al., 1994; Hossain and Yeasin, 2014). This can be used to help identify possible risk factors, such as if an individual may not be fully processing information being delivered by a complex or sensitive system.

A range of techniques have been used to record eye movements for research since work began in the early 20th century, including methods such as attaching electrodes to the skin around the eye or using contact lenses with an embedded metal coil that can be used to detect eye movements (Poole and Ball, 2006). More recent technologies are less invasive and often involve use of an infra-red camera to infer point-of-regard from the reflection that is given from the cornea, which is the outermost layer of the eye. These cameras can be placed beneath or next to a computer monitor in a way that is unobtrusive. Mobile eye trackers operate using the same principles but are worn in the same manner as a pair of spectacles, which allows for the individual to navigate their environment in a naturalistic style (Cristina and Camilleri, 2018). In the case of cybersecurity this could for instance involve exploring what a social engineer pays attention to when entering the reception area of a company, such as the location of security cameras or the presence of a PC at the reception desk.

This technology has been used to understand user behavior in relation to phishing websites. These are fraudulent websites designed to appear as genuine website, such as for example an internet banking page. Research suggests that only a quarter of people can reliably discriminate between genuine websites and fraudulent websites more than 75% of the time (Iuga et al., 2016). Technological approaches such as spam filters and machine learning may mitigate some of the risk posed by phishing attacks, but it has been argued that technology alone cannot completely prevent this issue (Pfeffel et al., 2019). This highlights the need to better understand the mechanisms behind a successful phishing attack. By using eye tracking it is possible to explore what factors predict whether someone will be tricked by a phishing website, by considering the interaction between the structure of the website and what the person looks at, or indeed fails to look at (Miyamoto et al., 2014). This has been used for example to understand how and if users pay attention to web browser security indicators, such as the Firefox Mozilla SSL certificate (Sobey et al., 2008). Research in this areas has revealed several techniques that have been identified in such phishing websites (Darwish and Bataineh, 2012) each of which can be researched through the use of eye tracking (Miyamoto et al., 2015). This includes the use of similar or related domain names (e.g., replacing a “w” in a website address with a “vv”), the use of high quality of animations to give fraudulent websites a professional feel and the presentation of fake Digital Certificates.

Further uses of eye tracking in cybersecurity have become evident as the research field and technology have continued to develop. For instance it has been demonstrated that the technology can be used to change risky behaviors, such as for example by preventing a user from continuing with use of input forms in a website unless an eye tracker has determined that the individual has looked at the address bar (Miyamoto et al., 2014). Similarly, eye trackers can be used to detect anomalous user behavior. The way in which an individual navigates a system that they are highly familiar with will be different from someone who is less familiar with a system: from work on expertise in visual processing (Miyamoto et al., 2015). Eye movement patterns are highly specialized and detectable when viewing scenes and objects that we are experts at processing (Liversedge et al., 2013). This difference in style will be reflected in eye movements, and could be used as a basis for detecting illicit behavior (Biedert et al., 2012). Recently it has been claimed that eye tracking machines themselves may not be necessary, and that webcams built into phones, laptops and tablets may be sufficient (Krafka et al., 2016) for many of the purposes discussed here. If this is the case, then it removes a major barrier for the adoption of eye tracking related cybersecurity measures in real life situations such as the workplace. As has been noted any technology that is used to protect users from cyber-attack is most effective then it is unobtrusive (Miyamoto et al., 2015).

Whilst there has been research using eye trackers to understand engagement with phishing website there is less research applying this technology to phishing emails (Baki et al., 2017), which are one vector through which targets may be directed to a phishing website in the first instance. There are recommendations made to the public by various organizations around what is likely to denote something as being a phishing email, such as the National Cyber Security Centre advice to look for misspellings, the use of urgency and the use of threatening language (National Cyber Security Centre, 2020), which reflects the typical features of phishing emails identified in the literature (Pfeffel et al., 2019). There is a lack of academic research that has explored the relationship between these features, including how trustworthy such emails are rated and how eye-movements may moderate this relationship. To address this we conducted an exploratory study in which we created phishing emails that employed characteristics and techniques evident in phishing emails, including the presence of misspellings in the sender address, the mention of financial information, the use of threatening language (for example that legal action will be taken if an email is not responded to) and the use of urgency.

## Materials and Methods

### Participants

Twenty-two psychology undergraduates (90% female, age range = 18 to 26, mean age = 20.29) were recruited from a sample responding to an online advertisement. Participants were awarded course credits for their participation.

### Design

A within-subjects design was employed in which participants were shown emails that either did or did not include a phishing indicator. There were four types of phishing indicator: financial information, urgency, misspelling, and threat. Stimuli were presented in a random order. Eye-tracking measures used were total dwell time, mean fixation count (denoting interest in a particular content), number of regressions (revisits, indicating that the item required further scrutiny because it drew attention), mean glance duration (denoting depth of processing), entry time and entry sequence (the time and fixation number that an area was attended to, denoting ease of attentional capture).

### Materials

Thirty-two emails were constructed based upon typical phishing type emails. These were split between the four types of email (misspelling, threatening, urgency, and financial) with four variations of each email type and either containing the phishing email indicator or not. This reflected the elements identified in public guidance from the National Cyber Security Centre on what may be an indicator that an email is a phishing one (National Cyber Security Centre, 2020). These emails were created by the researchers to be relevant to the study sample in terms of names of local organizations and national companies that the email purported to have been sent from. A publicly accessible database of suspected phishing emails^1^ was used to guide the creation of the study materials to ensure that these were consistent with phishing emails currently in circulation. The phishing emails created in this study were simple word documents structured according to emails in Microsoft Outlook. These contained a from line with email address, a subject line, the main content with roughly four sentences of text and a by-line.

Areas of interest (AOIs) were mapped onto the emails *post hoc* in BeGaze. This software is used to specify the areas of an image upon which the analysis will be based. These areas of interest were non-overlapping and focused on the core textual information. AOIs were: the email address, subject line, the addressee, the instruction line, any detail (hyperlinks, tracking numbers), and the phishing indicator (financial information, misspelling, threat, and urgency). AOIs were invisible to participants.

Stimuli were presented on an SMI RED 500 eye-tracker with in-built infrared cameras detecting eye movements. The screen was a 22-inch high-resolution LCD. Eye movements were recorded at 500 Hz with an accuracy of 0.4° of visual angle using SMI iView.

### Procedure

Piloting was conducted with a sample of 8 postgraduate research students. The purpose of this was to test the feasibility of using the eye tracker facilities for the intended purposes of the study. These trials involved participants using the same equipment to view examples of phishing emails. These emails were not split by type and participants were not asked to provide any rating of the emails. No technological or methodological problems were identified during this piloting phase.

Once piloting was completed the main study commenced. After providing informed consent, participants were told that they would be viewing a series of emails and that they would be asked to give a rating of how trustworthy they felt each email appeared to be. Participants’ eyes were then calibrated to the eye tracker using the standard in-built 9-point calibration procedure. Following calibration, the eye tracking was validated, to ensure consistent and accurate tracking. Validation consisted of the standard SMI calibration and validation procedure. Participants were requested to follow a ball around to 7-pseudo random locations around the screen. Calibration was considered successful if the eyes were calibrated within 1° of visual angle. If calibration failed, the participant was recalibrated once, otherwise they were removed from the analysis. The calibration was validated using the default procedure – participants eyes fixated on the center of the screen and if this was recording accurately, the trial proceeded. This validation was repeated after every 13 trials. Following this, participants began the experimental task. There were 32 identical trials.

In each trial, participants saw a blank fixation screen lasting 500 ms. Following this, participants saw the email. For each, participants were tasked with reading the email ready to rate it for trustability. Each email was on screen for 10 s. This time was chosen to represent the rather short amount of time that is devoted to reading each email that individuals receive (Hart, 2017). After the email, participants were given the rating screen, in which they were visually asked to rate how trustworthy the preceding email was on an 8-point Likert-type scale with the anchor points “Not at all trustworthy” and “Highly trustworthy.” Participants notified the researcher verbally of their choice, who then entered their answer into a numerical keypad. This was done to avoid unnecessary head movements by the participant. Following completion of all trials, participants were thanked and debriefed.

### Analysis Protocol

We assessed first whether the AOI containing the phishing indicator was scanned. To assess this, we analyzed whether the phishing indicator AOI was scanned more than would be expected by chance. For this, we ran a series of Bonferroni-corrected (α = 0.0125) one-subjects *t*-tests (two-tailed) comparing to a chance value for the region (which was based on the AOI size relative to the size of the screen). Secondly, we analyzed the amount of scanning to the other AOIs with and without the phishing indicator. Because the AOIs filled proportionally less of the screen in the phishing indicator present conditions, we area-normalized the AOIs by calculating the proportion of the screen that the AOIs occupied in each screen.

Our secondary analysis concerned which type of phishing indicator was most detectable. This was assessed with a one-way-ANOVA on the area-normalized phishing AOIs. For all analyses, the assumptions of parametric data were tested: Whenever Mauchley’s test of sphericity was significant, the Huynh-Feldt correction was applied to the degrees of freedom. If tests of normality were violated, a non-parametric test was used. For *post hoc* tests, the *p*-values were adjusted for multiple comparisons.

## Results

Figures 1–4 show examples of emails with the four types of phishing indicator. Figures 5–12 show a series of heat maps indicating where participants scanned images, split into the four pairs of emails either with or without the phishing email indicator (financial information, misspelling, threat, and urgency). We present an example of each category of phishing email with and without the phishing content for ease of understanding.

**FIGURE 1:**
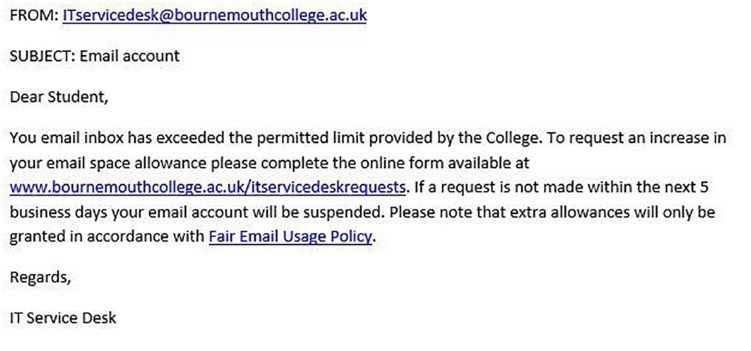
Example of email with urgency indicator.

**FIGURE 2:**
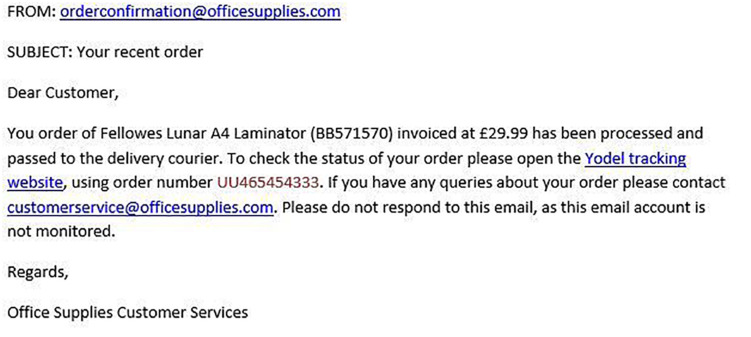
Example of email with financial information indicator.

**FIGURE 3:**
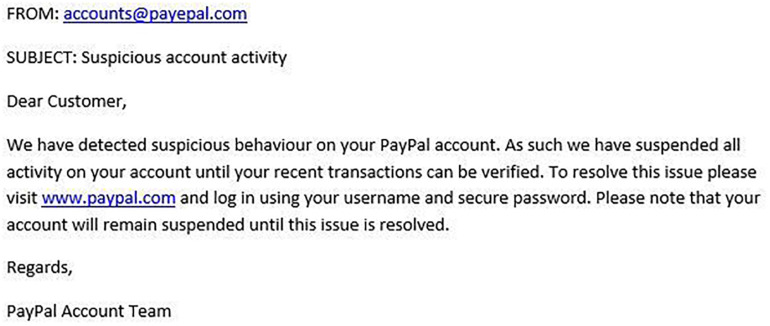
Example of email with misspelling indicator.

**FIGURE 4:**
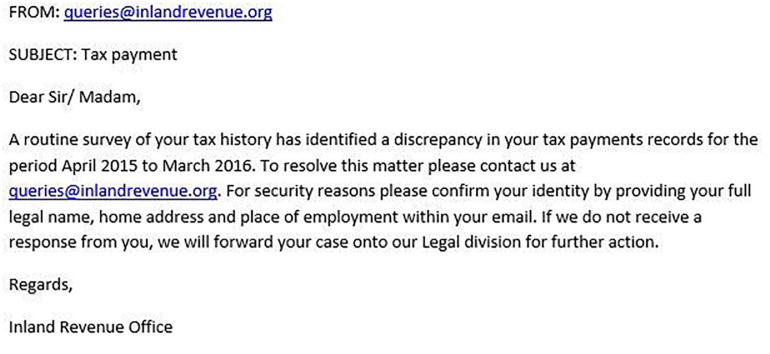
Example of email with threat indicator.

**FIGURE 5:**
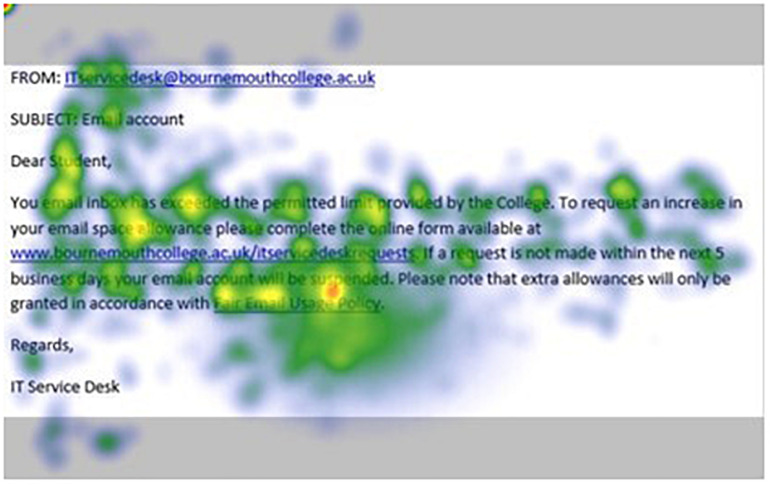
Heat maps averaged across all participants for email with urgency indicator.

**FIGURE 6:**
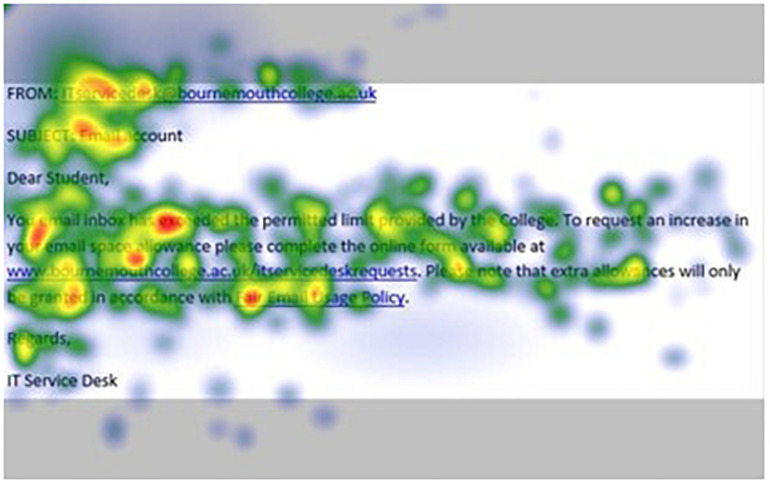
Heat maps averaged across all participants for matched email without urgency indicator.

**FIGURE 7:**
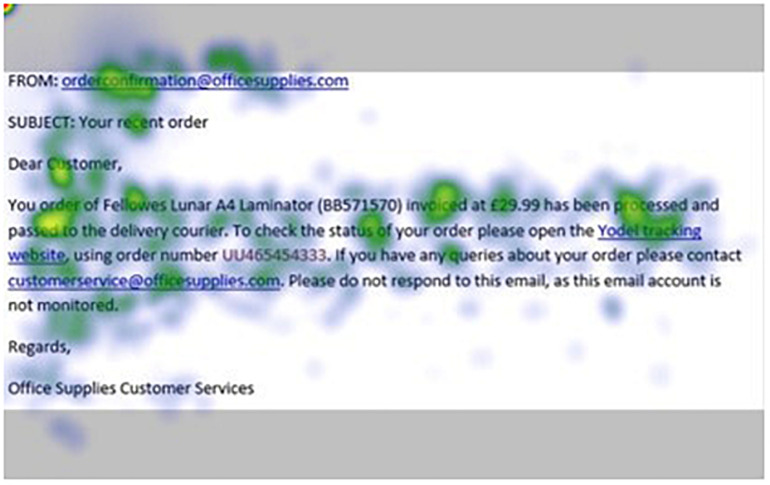
Heat maps averaged across all participants for email with financial information indicator.

**FIGURE 8:**
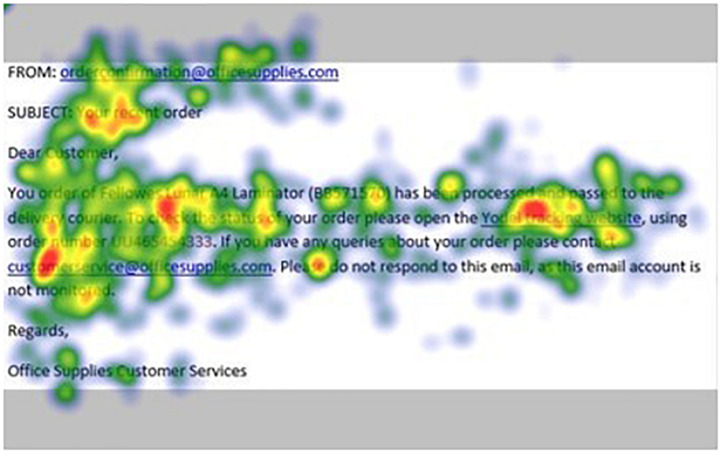
Heat maps averaged across all participants for matched email without financial information indicator.

**FIGURE 9:**
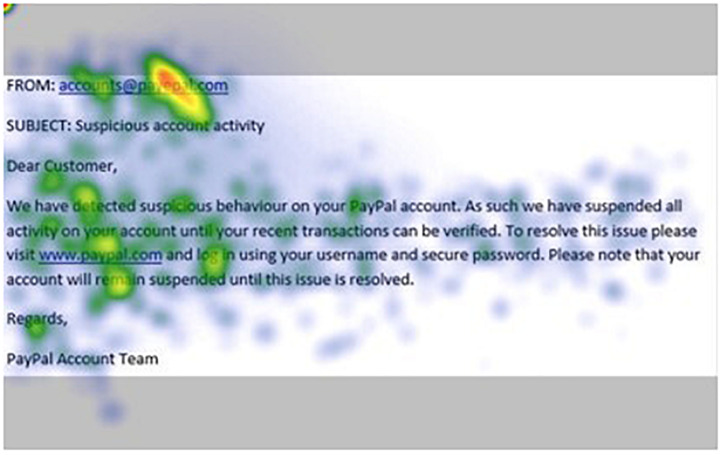
Heat maps averaged across all participants for email with misspelling indicator.

**FIGURE 10:**
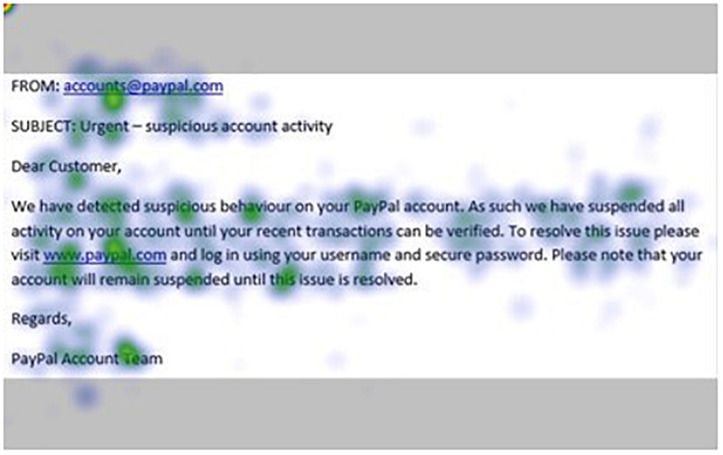
Heat maps averaged across all participants for matched email without misspelling indicator.

**FIGURE 11:**
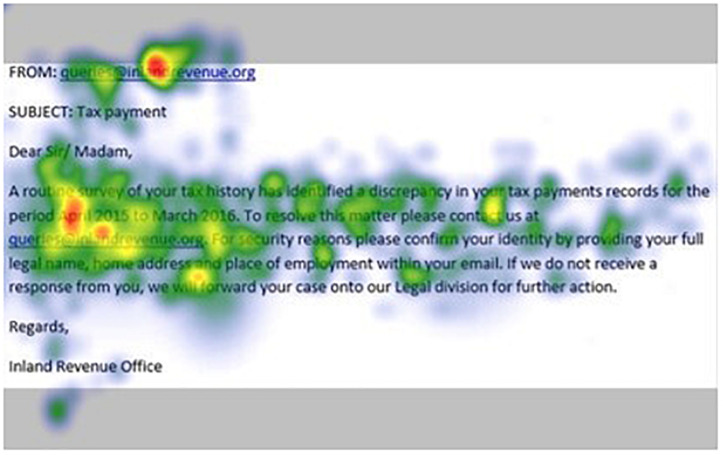
Heat maps averaged across all participants for email with threat indicator.

**FIGURE 12:**
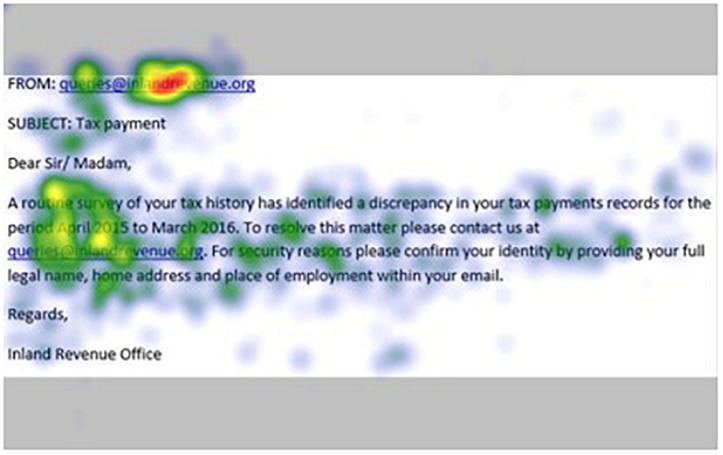
Heat maps averaged across all participants for matched email without threat indicator.

Our analysis protocol was applied, and summary statistics for the one-sample *t*-tests are shown in Table 1. Specifically, these results show that while the phishing AOIs were scanned (denoted by fixation count) and revisited (regression count) more frequently with more intense scanning (glance duration) than one would expect by chance, the total duration of scanning (dwell time) was less then would be expected by chance. In other words, less time was spent viewing the phishing indicators even though they required greater attentional resources paid to them.

**TABLE 1:** Mean (and standard error of the mean) for total dwell time (area-normalized ms), mean fixation count, number of regressions, and mean glance duration (ms), with one-sample *t*-value (df), and significance level.

		Mean	*t*-value	*p*-value
Financial information	Dwell time (*ms*)	159 (23)	−9.06 (20)	<0.001
	Fixation count	1.40 (0.40)	13.64 (14 )	<0.001
	Regression count	0.22 (0.09)	2.51 (14)	=0.025
	Glance Duration (*ms*)	93 (12)	7.87 (19)	<0.001
Misspelling	Dwell time (*ms*)	479 (101)	−3.03 (20)	=0.007
	Fixation count	3.31 (0.63)	5.25 (17)	<0.001
	Regression count	1.55 (0.44)	3.50 (17)	=0.003
	Glance Duration (*ms*)	163 (20)	8.33 (20)	<0.001
Threatening content	Dwell time (*ms*)	629 (50)	−74.48 (20)	<0.001
	Fixation count	3.18 (0.90)	15.83 (19)	<0.001
	Regression count	1.47 (0.19)	7.79 (19)	<0.001
	Glance Duration (*ms*)	104 (37)	12.91 (20)	<0.001
Urgency content	Dwell time (*ms*)	709 (78)	−49.68 (20)	<0.001
	Fixation count	3.50 (0.45)	7.72 (19)	<0.001
	Regression count	1.46 (0.31)	4.78 (19)	<0.001
	Glance Duration (*ms*)	104 (44)	10.94 (20)	<0.001

*Degrees of freedom are lower due to missing values for some participants.*

**FIGURE 13:**
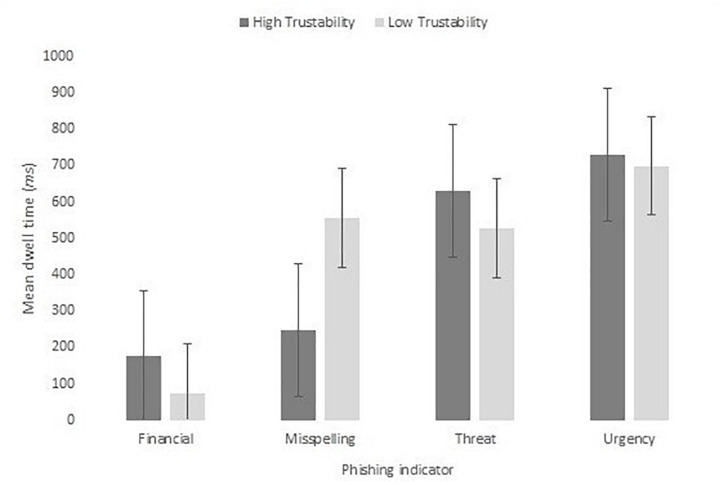
Mean dwell time to the scam item for high and low trustability emails split by indicator type.

Our second analysis focused on exploring whether the presence of the phishing indicator affected the scanning of the other content. The presence of each type of phishing indicator did not significantly affect normalized dwell time, *F*(1,20) = 0.06, *MSE* = 28486, *p* = 0.813, η_p_^2^ < 0.01. Figure 13 shows the mean dwell duration to each of the area-normalized AOIs for those with and without the phishing content.

Our final analysis concerned which type of phishing indicator would be more noticeable. Table 2 shows the means for each eye-tracking measure. Phishing indicator type affected: total dwell time, *F*(3,39) = 4.98, *MSE* = 800312348, *p* = 0.031, η_p_^2^ = 0.28, fixation count, *F*(3,39) = 6.30, *MSE* = 20.29, *p* = 0.014, η_p_^2^ = 0.33, regression count, *F*(3,39) = 6.72, *MSE* = 0.95, *p* = 0.003, η_p_^2^ = 0.34, glance duration, *F*(3,57) = 5.89, *MSE* = 68.76, *p* = 0.004, η_p_^2^ = 0.24, entry time, *F*(3,39) = 8.24, *MSE* = 0.5184111, *p* = 0.003, η_p_^2^ = 0.34, and sequence, *F*(3,39) = 4.72, *MSE* = 1.91, *p* = 0.024, η_p_^2^ = 0.27.

**TABLE 2:** Mean (and standard error of the mean) for total dwell time (area-normalized ms), mean fixation count, number of regressions, mean glance duration (ms), entry time (ms), and sequence.

	Financial phishing indicator	Misspelling phishing indicator	Threatening phishing indicator	Urgency phishing indicator
Total dwell time (ms)	159 (23)	479 (101)	630 (50)	709 (78)
Fixation count	1.43 (0.11)	2.59 (0.44)	3.35 (0.15)	3.52 (0.51)
regressions count	0.24 (0.09)	0.92 (0.24)	1.60 (0.23)	1.22 (0.24)
Glance duration (ms)	93 (12)	165 (20)	104 (9)	105 (10)
Entry time (ms)	3712 (529)	4882 (1474)	1603 (241)	1395 (247)
Sequence	3.52 (0.28)	3.23 (0.36)	4.16 (0.34)	4.56 (0.21)

Specifically, financial indicators were viewed for less time than threat indicators (mean difference = 469, *p* = 0.015, *r* = 0.79) and urgency indicators (mean difference = 550, *p* = 0.019, *r* = 0.72). Further, they were viewed less frequently with less regressions than threatening indicators (mean difference_fixation count_ = 2.09, *p* < 0.001, *r* = 0.61, mean difference_regression count_ = 1.36, *p* < 0.001, *r* = 0.97) and urgency indicators (mean difference_fixation count_ = 1.92, *p* < 0.001, *r* = 0.89, mean difference_regression count_ = 0.92, *p* < 0.001, *r* = 0.59). Glance duration was shorter for threat indicators than financial indicators (mean difference = 60.74, *p* = 0.044, *r* = 0.40). Threat and urgency indicators were viewed earlier than misspelling indicators (threat: mean difference_entry time_ = 1706 *p* < 0.001, *r* = 0.56, mean difference_sequence_ = 0.93, *p* < 0.001, *r* = 0.33; urgency: mean difference_entry_ time = 2855, *p* < 0.001, *r* = 0.74 and mean difference_sequence_ = 1.33, *p* = 0.011, *r* = 0.52).

**FIGURE 14:**
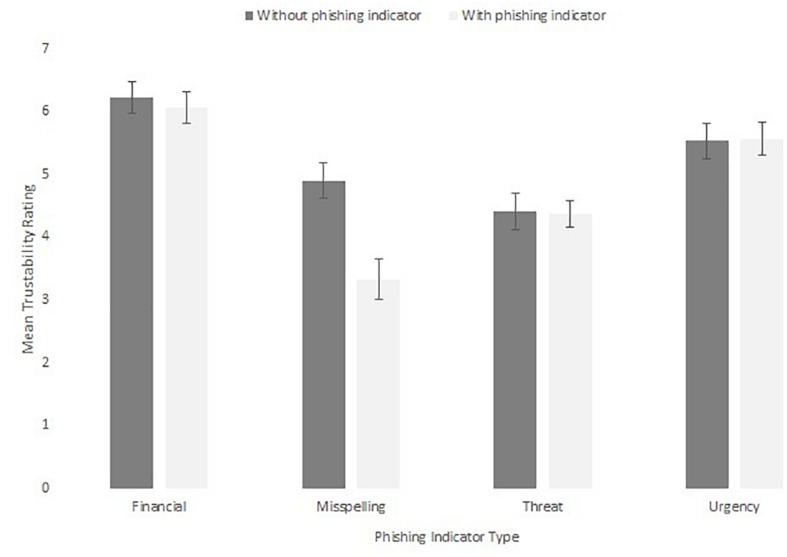
Mean trustability ratings for each phishing indicator.

A further set of analyses were run on the trustability ratings, shown in Figure 14. These were subjected to a 2 × 4 within-subjects ANOVA. This revealed a main effect of phishing indicator, *F*(3,60) = 25.63, *MSE* = 1.50, *p* < 0.001, η_p_^2^ = 0.58. Emails with misspelling and threatening phishing indicators were rated as less trustworthy than financial (mean difference = 2.02, *p* < 0.001, *r* = 0.76, mean difference = 1.75, *p* < 0.001, *r* = 0.70) and urgency (mean difference = 1.44, *p* < 0.001, *r* = 0.62, mean difference = 1.16, *p* = 0.002, *r* = 0.53) scams. There was also a main effect of presence of phishing indicator, *F*(1,20) = 10.87, *MSE* = 0.74, *p* = 0.004, η_p_^2^ = 0.35, in which phishing indicators present emails (5.27, *SE* = 0.21) were rated as less trustworthy than emails without phishing indicators (4.83, *SE* = 0.16). However, these effects interacted, *F*(3,60) = 9.45, *MSE* = 0.97, *p* < 0.001, η_p_^2^ = 0.32. The interaction was revealed by the effect of phishing indicator presence only being significant for the misspelling scam, *t*(20) = 4.05, *p* = 0.001, *r* = 0.50, and not for the other types of scams (smallest *p* = 0.397).

**FIGURE 15:**
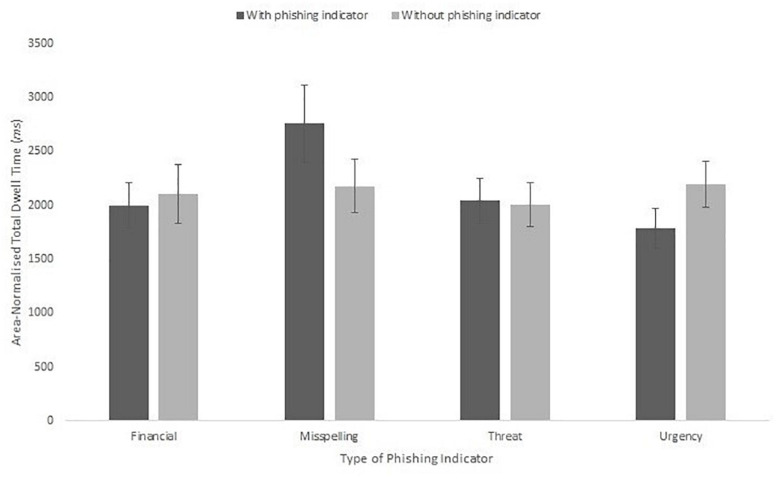
Mean area-normalized total dwell time split by indicator presence and absence.

Finally, we assessed whether the trustability rating influenced the amount of scanning to the phishing indicators of the emails. We used two protocols to assess this. In the first we ran a series of correlations between the dwell time for each email type and the trustability rating given. None of these correlations were significant: financial phishing indicators, *r*(19) = 0.20, *p* = 0.385; misspelling, *r*(19) = −0.41, *p* = 0.063; threat, *r*(19) = 0.10, *p* = 0.659; and urgency, *r*(19) = −0.26, *p* = 0.263. In the second, we analyzed whether dwell time to the phishing indicator item was different for emails rated as trustable (scoring higher than 4) compared to those rated as untrustable (rated 4 or lower) split by type of phishing indicator, shown in Figure 15. This analysis was done by-item. The resulting 4 × 2 mixed ANOVA showed no significant effect of trustability, *F*(1,12) = 0.06, *MSE* = 45904, *p* = 0.811, η_p_^2^ = 0.01, nor an interaction with phishing indicator type, *F*(1,12) = 1.68 *MSE* = 45904, *p* = 0.223, η_p_^2^ = 0.30.

## Discussion

The results of the study were notable in several ways. Participants spent less time overall looking at indicators of phishing than they would be expected to by chance. In addition, the presence of phishing indicators did not significantly impact on how much time is spent looking at the rest of the email. Overall, this may suggest that individuals require little processing time to recognize elements that relate to phishing. The phishing variants of each email were also rated as being less trustworthy than the non-phishing variants, suggesting that participants have some ability to recognize that the selected features are associated with fraudulent emails. Yet there was no statistically significant association between the trustworthiness rating and the total scanning time for the phishing indicators within the emails. As such whilst emails with phishing indicators were rated as less trustworthy than those without, this does not appear to be explained by how much time is spent attending to those phishing indicators. This makes it unclear whether the features of phishing emails that would appear to be designed to capture attention, exploit heuristics and invoke a cognitive miser style of processing are achieving this. An interpretation of this could be that the relationship between the presence of features related to phishing emails and how trustworthy that email is seen to be is more complex than expected. Similar unexpected, complex and inconsistent results have been found in relation to susceptibility to phishing emails and other factors including personality, knowledge of computers and gender (Kleitman et al., 2018).

Other aspects of the results were more in keeping with previous research. For instance, it was noted that participants would tend to look first at phishing indicators relating to urgency and threats before looking at misspellings and financial information. This could be a reflection of survival information bias (Nairne, 2010), in which individuals place priority on processing information that may relates to their well-being. Emails containing misspelling were also rated as being less trustworthy than the other emails, which may be due to the presence of misspelling being a more categorical factor than the use of urgency or threatening language, which are open to interpretation. Financial phishing email indicators were associated with the least frequent number of fixations and the least amount of overall dwell time, as compared to the phishing indicators in the misspelling, urgency, and threat email variations. Emails with financial phishing indicators were also rated as being more trustworthy than emails with misspelling or threat phishing indicators. This suggests that the inclusion of financial information within phishing emails has a lower impact of how that email is processed and to what degree it is trusted.

There were limitations to this study. A relatively small sample size was used, although this is not atypical when compared to other eye-tracking studies (Tecce et al., 1998; Libben and Titone, 2009; Choi et al., 2017). While the sample size was consistent with previous eye-tracking research, it is not sufficient to explore individual variability in how well eye movements predict ability to spot phishing emails. Further recruitment of participants was not possible due to constraints caused by the COVID-19 situation.

The participants consisted of a narrow demographic from a single geographical location. The sample was also predominantly female. There is no evidence of gender differences in eye movements (Klein and Ettinger, 2019) and a lack of consistent research on the role of gender in phishing email susceptibility (Kleitman et al., 2018). Nevertheless, having a more diverse sample may help identify if there are certain types of phishing email that are more impactful on different demographic groups. Due to the limited research in this area there was also a lack of baseline evidence to use to inform the creation of phishing email materials. Examples of phishing emails available on websites such as www.phishtank.com are not ideally suited to experimental designs, as they often include conflation of different phishing techniques, such as a combination of threat and urgency. We opted to create our own stimuli in this study to reduce the influence of such possible confounders, however, it is difficult to do so completely whilst keeping the stimuli realistic. Further refinement of these stimuli may also help clarify the relationship between content and how phishing emails are read and judged. Finally, we note that asking participants to provide a trustworthiness rating of the stimuli may have alerted them that the study related to phishing emails. As demonstrated by Parsons et al. (2015) participants may be more successful at identifying phishing emails when they are aware in advance that they may be about to do so.

The results of this study demonstrate some important points. It provides evidence that eye tracking technology can be used to determine whether people look at the common indicators of phishing emails, and also inform us on the order in which these are attended to. In doing so it also demonstrated some unexpected patterns, including that individuals look at phishing indicators more frequently than would be expected by chance but, counterintuitively, spend less overall time doing so than would be expected by chance. Building upon this research may provide more avenues for the understanding and mitigation of the serious threat that phishing emails pose to cybersecurity.

## Data Availability Statement

The datasets generated for this study are available on request to the corresponding author.

## Ethics Statement

The studies involving human participants were reviewed and approved by the Science, Technology & Health Research Ethics Panel, Bournemouth University. The patients/participants provided their written informed consent to participate in this study.

## Author Contributions

JM contributed to the content relating to phishing emails and social engineering. PH contributed to the content on eye-tracking technology and led the analysis. Both authors contributed to the article and approved the submitted version.

## Conflict of Interest

The authors declare that the research was conducted in the absence of any commercial or financial relationships that could be construed as a potential conflict of interest.

## Footnotes

1 www.phishtank.com
